# Targeted re-sequencing on 1p22 among non-syndromic orofacial clefts from Han Chinese population

**DOI:** 10.3389/fgene.2022.947126

**Published:** 2022-08-17

**Authors:** Mu-Jia Li, Jia-Yu Shi, Bi-He Zhang, Qian-Ming Chen, Bing Shi, Zhong-Lin Jia

**Affiliations:** ^1^ State Key Laboratory of Oral Diseases and National Clinical Research Center for Oral Diseases, West China Hospital of Stomatology, Sichuan University, Chengdu, China; ^2^ Department of Cleft Lip and Palate, West China Hospital of Stomatology, Sichuan University, Chengdu, China; ^3^ Division of Growth and Development and Section of Orthodontics, School of Dentistry, University of California, Los Angeles, Los Angeles, CA, United States

**Keywords:** 1p22, targeted re-sequencing, association analysis, LCLO, RNA sequencing

## Abstract

Rs560426 at 1p22 was proved to be associated with NSCL/P (non-syndromic cleft lip with or without the palate) in several populations, including Han Chinese population. Here, we conducted a deep sequencing around rs560426 to locate more susceptibility variants in this region. In total, 2,293 NSCL/P cases and 3,235 normal controls were recruited. After sequencing, association analysis was performed. Western blot, RT-qPCR, HE, immunofluorescence staining, and RNA sequencing were conducted for functional analyses of the selected variants. Association analysis indicated that rs77179923 was the only SNP associated with NSCLP specifically (*p* = 4.70E-04, OR = 1.84), and rs12071152 was uniquely associated with LCLO (*p* = 4.00E-04, OR = 1.30, 95%CI: 1.12–1.51). Moreover, *de novo* harmful rare variant NM_004815.3, NP_004806.3; c.1652G>C, p.R551T in *ARHGAP29* resulted in a decreased expression level of *ARHGAP29*, which in turn affected NSCL/P-related biological processes; however, no overt cleft palate (CP) phenotype was observed. In conclusion, rs12071152 was a new susceptible variant, which is specifically associated with LCLO among the Han Chinese population. Allele A of it could increase the risk of having a cleft baby. Rs77179923 and rare variant NM_004815.3, NP_004806.3; c.1652G>C, p.R551T at 1p22 were both associated with NSCLP among the Han Chinese population. However, this missense variation contributes to no overt CP phenotype due to dosage insufficiency or compensation from other genes.

## Introduction

Non-syndromic cleft lip with or without the palate (NSCL/P), one of the most common orofacial clefts, has an average prevalence of 1/1,000 live births worldwide, with a relatively high prevalence among Asians (Croen et al., 1998; Tolarova and Cervenka, 1998; Mossey and Modell, 2012). The affected kids usually suffer from a number of problems related to clefts, such as speech, hearing, and psychological disorders (Lewis et al., 2017). It is necessary for them to receive coordinated multidisciplinary care that lasts from the stage of infant to adulthood, which imposes a heavy financial burden on their families.

NSCL/P is a complex disorder, with genetic and environmental factors and their interplay involved (Dixon et al., 2011; Rahimov et al., 2012; Worley et al., 2018). However, genes play a dominant role (Grosen et al., 2010; Dixon et al., 2011; Rahimov et al., 2012; Baldacci et al., 2018). Thus, lots of studies have been designed to shed light on the susceptibility genes or loci for NSCL/P, among which genome-wide association studies (GWASs) have identified an unprecedented number of genetic variants associated with it, and to date, over 40 risk loci for NSCL/P have been identified (Leslie and Marazita, 2013; Lin-Shiao et al., 2019). However, those findings only account for about 20% estimated heritability of NSCL/P (Beaty et al., 2016; Lin-Shiao et al., 2019); the missing heritability is partially attributed to the strict significance threshold of GWAS, which leads to the failed detection of that single-nucleotide polymorphism (SNP) with modest effect (Manolio et al., 2009; Tam et al., 2019); in addition, those risk loci identified by GWAS are usually driven by associated genetic variants due to linkage disequilibrium (Altshuler et al., 2008; Dickson et al., 2010), thus making it difficult to pinpoint the casual variants. Based on this, high-depth sequencing targeted at those risk loci is a cost-effective method to identify variants with larger effect sizes that are missed by GWAS, and this would also facilitate the discernment of casual variants (Manolio et al., 2009; Sazonovs and Barrett, 2018).

1p22, which contains rs560426, was initially identified as one of the risk loci for NSCL/P because of the statistically significant association between rs560426 and NSCL/P via GWAS (Beaty et al., 2010). Our previous study indicated that rs560426 was significantly associated with NSCL/P among the Han Chinese population, which further conferred susceptibility to 1p22. Rs560426 is located in *ABCA4* gene, which is surely excluded from the candidate susceptibility genes in 1p22 due to its expression restricted to the retina (Beaty et al., 2010). Leslie et al. (2012) identified several rare variants that were associated with NSCL/P in *ARHGAP29*, which is adjacent to *ABCA4* and expressed in the developing face. Therefore, *ARHGAP29* was highly suspected as a susceptibility gene of NSCL/P in 1p22. From then on, a surge of studies focused on 1p22, and plenty of rare variants in *ARHGAP29* were identified in multiple ethnicities (Leslie et al., 2012; Butali et al., 2014; Chandrasekharan and Ramanathan, 2014; Letra et al., 2014; Gowans et al., 2016; Savastano et al., 2017).

In this study, we aim to conduct a deep screening targeting the 1p22 locus to fully dig into susceptibility SNPs or indels through bioinformatics, statistics analysis, and functional experiments, hoping to identify more susceptibility variants at this locus for NSCL/P among the Han Chinese population.

## Materials and methods

### Sample collection and ethics statement

In total, 159 NSCL/P cases were included in the deep sequencing phase of our study, whereas 542 controls’ WGS data with an average coverage of 39.89 was downloaded from the Novogene internal database (http://www.novogene.com/); 2,134 NSCL/P (1047 NSCLO and 1087 NSCLP) and 2,693 normal controls from West China Second University Hospital, Sichuan University, were recruited in the replication phase. Cases were collected between 2016 and 2018 from the Cleft Lip and Palate Surgery Department of West China Hospital of Stomatology, Sichuan University. All the participants were self-recognized as the Han Chinese and denied family history as well as other congenital diseases, therein, the phenotype of the patients was assessed by both physicians and geneticists. More details of samples are shown in Supplementary Table S1.

Our study abides by the STOBE (Strengthening the Reporting of Observational Studies in Epidemiology) guidelines and was approved by HEC (the Hospital Ethics Committee) of West China Hospital of Stomatology. All individuals voluntarily joined this study with informed consent (WCHSIRB-D-2016-012R1).

### Targeted region deep sequencing

DNA was extracted from peripheral blood of each sample by the salting-out method. After quality control, 1.0 μg of each DNA sample was enriched by using Agilent SureSelectXT Custom kit. Then, sequencing was conducted on the Illumina Hiseq X Ten platform to get paired-end 150bp reads by Novogene (China). The sequenced region was selected around rs560426 (GRCh37/hg19, chr1:94,453,779 to 94,739,314) based on the LD structure in CHB/JPT HapMap project.

### Bioinformatics analysis

After removing adapter-related reads, N-containing reads, and low-quality reads, the clean sequence data were mapped to the human genome GRCh37/hg19 by Burrows–Wheeler Aligner (BWA) software (Li and Durbin, 2009). Then, 943 single nucleotide variants (SNPs) and 390 insertion/deletions (In/Dels) were identified by the Sequence Alignment Map (SAM tools) (Li et al., 2009) and merged by VCF (variant call format) tools (version 0.1.13) (Danecek et al., 2011). Later, variants were annotated by ANNOVAR (version 201707) (Wang et al., 2010), followed by function prediction via SIFT (Ng and Henikoff, 2003), v1.3 CADD (Kircher et al., 2014), Polyphen-2 (http://genetics.bwh.harvard.edu/pph2/) (Adzhubei et al., 2013), and MutationTaster (http://www.mutationtaster.org/) (Schwarz et al., 2010).

### Statistical analysis

In the discovery phase, variants were categorized as either common or rare. Variants with MAF (minor allele frequency) ≥1% were referred to as common variants (they were Single nucleotide polymorphisms or SNPs), and case–control association analysis was performed after excluding SNPs that deviated from Hardy–Weinberg equilibrium (HWE). Three rare variants selected by three conditions were enrolled into burden analysis calculated by the R package SKAT: ① MAF <1% in the CHB population (Beijing Han Chinese population) and CHS population (Southern Han Chinese population) from 1000 Genomes Project database and Novogene internal database; ② MAF <0.001 in the Genome Aggregation Database (GnomAD); ③ at least two prediction tools suggested its harmfulness (SIFT, v1.3 CADD, Polyphen-2, and MutationTaster).

In the replication phase, SNP genotyping data were retrieved from two GWASs we have ever participated in (Sun et al., 2015; Huang et al., 2019). PLINK software (version 1.9) was used to perform the HWE test, calculate MAF, and perform a case–control association analysis for each SNP (Purcell et al., 2007). The threshold of *P*-value is 0.05/99 = 5.05E-04.

### Sanger sequencing

Three novel harmful rare variants, which were not reported in a public database, such as dbSNP (https://www.ncbi.nlm.nih.gov/SNP/) (Smigielski et al., 2000), 1000 Genome (https://www.internationalgenome.org/), ExAC (http://exac.broadinstitute.org) (version 0.3.1) (Lek et al., 2016), CADD (http://cadd.gs.washington.edu/snv) (Rentzsch et al., 2018), and HGMD (http://www.hgmd.org) (Stenson et al., 2009), were further validated in carriers and their parents by Sanger sequencing, and PCR primers for genomic sequence were designed using Primer 3 (https://bioinfo.ut.ee/primer3-0.4.0/) (Supplementary Table S2). Then, for amplification, a mixture of Taq polymerase enzyme, PCR primers, water, and DNA sample was prepared. The amplified DNA products were then sequenced using the ABI 3730 Sequencer and analyzed with Sequence Scanner v1.0.

### Cell culture and transient transfection

HEK-293T cell line was cultured in Dulbecco’s modified Eagle medium (DMEM) with 10% fetal bovine serum (PAN Biotech, Germany) and 1% Penicillin–Streptomycin Solution (Gibco, Foster City, CA, United States).

Full-length cDNA of *ARHGAP29* (NM_004815.3) was synthesized and sub-cloned into pcDNA3.1 plasmid, to which site-directed mutagenesis was applied and thus obtained pcDNA3.1-*ARHGAP29* ^*R551T*^ plasmid (GeneChem, China). Then, they were transfected into HEK-293T cells by using Lipofectamine 3000 (Invitrogen, Carlsbad, CA) according to the manufacturer’s instructions, respectively.

### Construction of the *Arhgap29* ^*R553T*^ mutant mouse model

The homology analysis of the amino acid sequences of human and mouse ARHGAP29 revealed that the 553rd amino acid of mouse ARHGAP29 was identical to the 551st amino acid of humans. Therefore, the CRISPR/Cas9 system was used to engineer a single base substitution mutation from G to C at the 1658th nucleotide of the cDNA of the *Arhgap29* gene in the C57BL/6J mouse, resulting in a change from arginine (R) to threonine (T) at the 553rd amino acid. This part of the experiment was conducted by Gempharmtech Biotechnology Company (China), from whom we acquired F1 heterozygous *Arhgap29* ^*R553T/+*^ mice for the subsequent experiments.

Due to the limited number of F1 heterozygous *Arhgap29* ^*R553T/+*^ mice, they were crossed to C57BL/6J wild-type mice to generate a sufficient number of heterozygous mice. After genotyping the offsprings, heterozygous *Arhgap29* ^*R553T/+*^ mice were chosen to be maintained. To be specific, 1–2 mm tail tissue was cut off from each mouse, from which DNA was extracted and amplified by PCR (Forward primer: CCACCACTTCTGTGGTGTCCTTG, reverse primer: CTACCCATGTTCTGCCTGTTGAG), both of which were completed using One Step Mouse Genotyping Kit (Vazyme, China). Sanger sequencing was then performed on those PCR products to confirm the genotype of each mouse.

After that, heterozygous *Arhgap29* ^*R553T/+*^ mice were crossed overnight, females were examined for the presence of a vaginal plug the next morning, and the day when the vaginal plug was observed was designated as embryonic day 0.5 (E0.5).

### RNA extraction

RNA was extracted from each group of HEK-293T cells using RNA-easy Isolation Reagent (Vazyme, China) 48 h after transfection. At E13.5, RNA was extracted from the secondary palate of homozygous *Arhgap29* ^*R553T/R553T*^ and wild-type mice. A total of 500 ng RNA was undertaken reverse transcription PCR to form cDNA by Takara PrimeScript kit.

### RNA sequencing

Using the BGISEQ-500 platform, RNA sequencing was performed on the cDNA library of *Arhgap29* ^*R553T/R553T*^ and wild-type mice (BGI, China). In each group, two biological replications were included. Using DEseq2 and the Gene Ontology (GO) database, differential gene expression analysis and annotation for the biological process of DEGs (differential expression genes) were conducted.

### Quantitative real-time qPCR

RT-qPCR was performed by using Takara TB Green Premix ExTaq. *GAPDH* was chosen as a reference gene, and primers are shown in Supplementary Table S3. Results were analyzed using the 2^−ΔΔCt^ method. Each of the three biological replications was accompanied by three technical replications. Statistical analysis was calculated by the unpaired two-tailed t-test in GraphPad Prism 8 software.

### Western blot

Furthermore, 48 h after transfection, after discarding the culture medium and washing with PBS, 250 μl of lysate was added to each well (containing 10 dsμl of PMSF per 1000 μl of RIPA) (Beyotime, China), carefully pipetted, and placed on ice for 10 min. Then, the lysate was collected and centrifuged at 10,000g for 3 min, the supernatant was diluted with ×5 loading buffer (Beyotime, China) and boiled for 10 min.

Subsequently, protein samples were separated by electrophoresis in agarose gels and transferred onto PVDF membranes, which were then blocked by 5% milk for 1 h and incubated with rabbit anti-human Arhgap29 antibody (Novus Biologicals, United States) at 4°C overnight, followed by incubation with anti-rabbit antibody (Proteintech, China) at room temperature for 1 h. At last, proteins were visualized by ECL substrate (Epizyme, China).

### Micro-CT scanning

Three homozygous *Arhgap29* ^*R553T/R553T*^ and wild-type mice were selected and their entire body bone tissues were scanned by Micro-CT. The X-ray tube voltage was set to 70 kV, and the current was 114 A. The reconstruction was performed with Mimics 21.0.

### HE and immunofluorescence staining

Embryos from E13.5 to E15.5 were fixed overnight in 4% paraformaldehyde and then fixed in paraffin. Serial paraffin sections of 7 μm were collected and deparaffinized in xylene and rehydrated with a range of ethanol concentrations. For regular histology, hematoxylin and eosin were used to stain tissue sections. For immunofluorescence, after heat-induced antigen retrieval, samples were blocked for 1 h with 5% bovine serum albumin in phosphate-buffered saline. The rabbit anti-human Arhgap29 antibody (Novus Biologicals, United States) was incubated overnight at 4°C. Following a PBST wash, rabbit IgG Alexa 488 (Abcam, United States) was applied for 1 h at room temperature, followed by another wash. Images were captured after mounting samples with DAPI.

## Results

### Rs77179923 was specifically associated with NSCLP

By targeted region sequencing, we detected a total of 943 single nucleotide variations (SNVs) and 390 In/Dels. Of them, 656 SNVs were recognized as common variants and recruited into case–control association analysis, whereas 3 rare variants were enrolled in burden analysis (data did not show any significance).

In the discovery phase, 99 of the 656 SNVs in our targeted region were identified to be potential susceptibility variants of NSCL/P with a *P*-value less than 0.05 (Supplementary Figure S1 and Supplementary Table S4). Subsequently, all the 99 SNPs were replicated among 1,626 NSCL/P cases and 2,255 controls. According to the significance threshold after multiple corrections, the SNPs with a *P*-value less than 5.05E-04 are associated with the replication phase.

**FIGURE 1:**
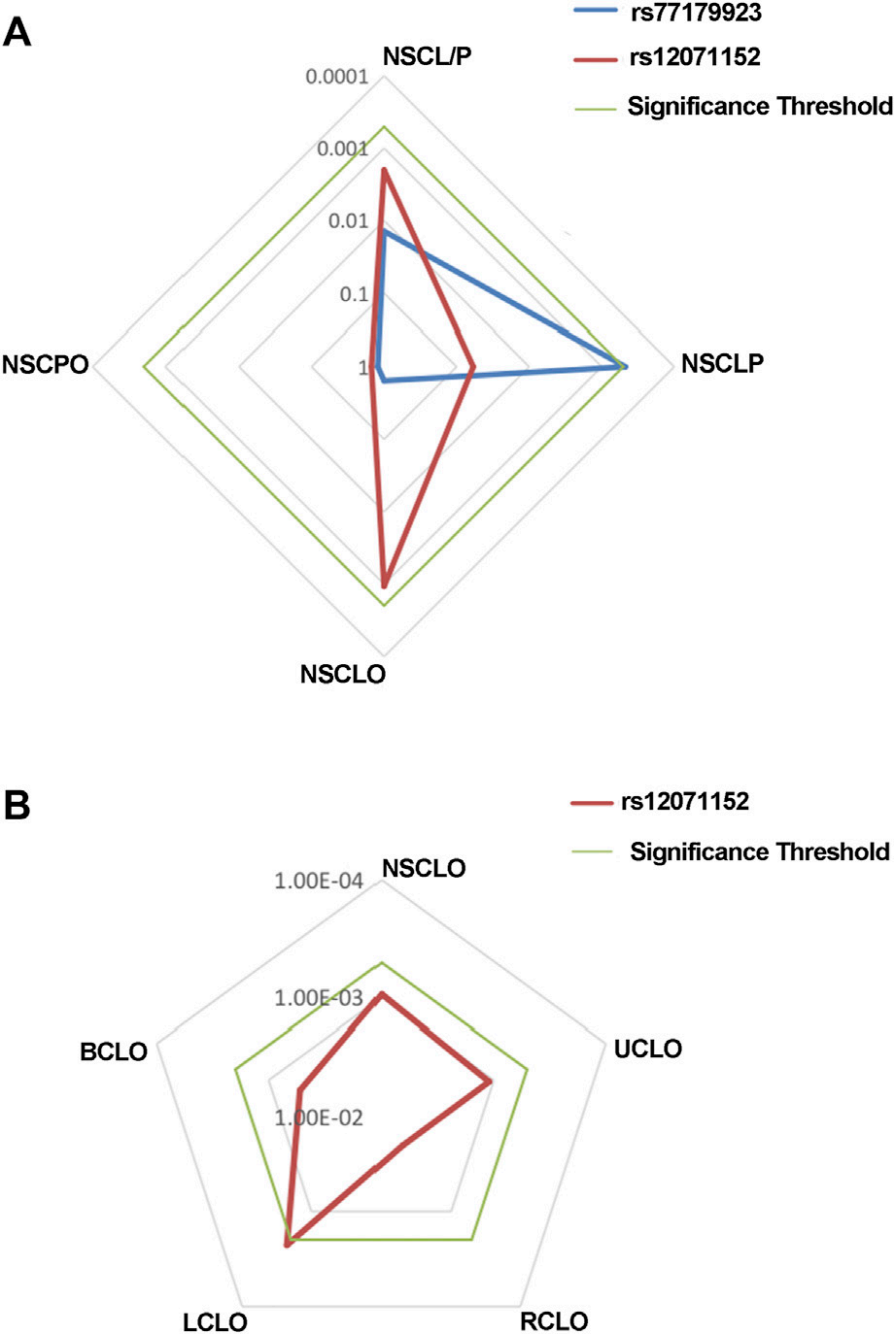
Radar chat for the replication of the association analysis. Log *P* value with base 10 were shown in the chat, while blue, red and green line indicate the result of rs77179923, rs12071152 and significance threshold respectively. Significance threshold in the replication phase is 5.05E-04, which is adjusted by multiple correction.

MAF and HWE (Supplementary Table S5) of the replicated SNPs were calculated, and those SNPs with MAF above 1% and *P*-value of HWE above 0.05 were recruited into the association analysis. Interestingly, we found that rs77179923 was specifically associated with NSCLP (*p* = 4.70E-04, OR = 1.84, 95%CI: 1.31–2.58), and its T allele was at risk for NSCLP, which indicated that the carries could have a higher risk to give birth a cleft baby. Rs12071152 was marginally associated with NSCLO (*p* = 9.40E-04, OR = 1.27, 95%CI:1.10–1.46). None of SNPs was identified to be associated with NSCL/P (Table1 and Figure 1A).

**TABLE 1:** Replication of the association analysis in 1p22.

SNP	A1	NSCL/P		NSCLP		NSCLO	
		*P*	OR (95%CI)	*P*	OR (95%CI)	*P*	OR (95%CI)
rs2282229	A	0.150	0.84 (0.67–1.06)	0.640	1.08 (0.78–1.49)	0.021	0.71 (0.54–0.95)
rs11165065	A	0.350	0.91 (0.75–1.11)	0.370	1.13 (0.87–1.47)	0.057	0.79 (0.62–1.01)
rs560426	C	0.430	1.05 (0.94–1.17)	0.730	1.03 (0.88–1.20)	0.550	1.04 (0.91–1.20)
rs77179923	**T**	0.013	1.47 (1.08–2.00)	**4.70E-04**	**1.84(1.31–2.58)**	0.640	1.13 (0.68–1.89)
rs12088309	C	0.050	1.11 (1.00–1.23)	0.190	1.10 (0.95–1.28)	0.066	1.13 (0.99–1.28)
rs2297636	C	0.077	1.10 (0.99–1.22)	0.700	1.03 (0.89–1.19)	0.018	1.17 (1.03–1.33)
rs12057375	T	0.043	1.13 (1.00–1.26)	0.140	1.13 (0.96–1.32)	0.110	1.12 (0.97–1.30)
rs3789434	C	0.050	1.12 (1.00–1.26)	0.170	1.12 (0.95–1.31)	0.110	1.12 (0.98–1.30)
rs4147810	G	0.042	1.13 (1.00–1.26)	0.140	1.13 (0.96–1.32)	0.100	1.13 (0.98–1.30)
rs2297635	A	0.048	1.12 (1.00–1.26)	0.170	1.12 (0.95–1.31)	0.110	1.12 (0.97–1.30)
rs3789438	T	0.040	1.13 (1.01–1.27)	0.160	1.12 (0.96–1.31)	0.093	1.13 (0.98–1.30)
rs11165079	T	0.340	0.94 (0.82–1.07)	0.580	0.95 (0.79–1.14)	0.340	0.93 (0.79–1.09)
rs11165080	G	0.300	0.93 (0.82–1.06)	0.560	0.95 (0.79–1.14)	0.300	0.92 (0.78–1.08)
rs1931570	T	0.340	0.94 (0.82–1.07)	0.600	0.95 (0.79–1.15)	0.340	0.93 (0.79–1.09)
rs1931566	G	0.340	0.94 (0.82–1.07)	0.590	0.95 (0.79–1.14)	0.330	0.92 (0.79–1.08)
rs12071152	A	0.002	1.19 (1.06–1.33)	0.060	1.16 (0.99–1.36)	9.40E-04	1.27 (1.10–1.46)

The table shows SNPs with *p* < 0.05 in the replication phase. A1, minor allele; SNP, single nucleotide polymorphism; NSCL/P, non-syndromic cleft lip with or without the palate; NSCLP, non-syndromic cleft lip with the cleft palate; NSCLO, non-syndromic cleft lip only. OR refers to odds ratio. 95%CI refers to 95% confidence interval. *P* refers to *P*-value for this test. The bold characters indicated the significant SNPs after multiple corrections (significant threshold is 5.05E-04).

### Rs12071152 was uniquely associated with LCLO

To further test if the 99 SNPs associated with sub-phenotypes of NSCLO, we divided NSCLO into BCLO (bilateral cleft lip only), UCLO (unilateral cleft lip only), RCLO (right cleft lip only), and LCLO (left cleft lip only). Intriguingly, we noticed that rs12071152 showed specific association with LCLO (*p* = 4.00E-04, OR = 1.30, 95%CI: 1.12–1.51); although the association between rs12071152 and NSCLO (*p* = 9.40E-04, OR = 1.27, 95%CI: 1.10–1.46) did not survive after multiple corrections, its association with BCLO (*p* = 0.002, OR = 1.28, 95%CI:1.10–1.49), RCLO (*p* = 0.005, OR = 1.24, 95%CI:1.07–1.45), and UCLO (*p* = 0.001, OR = 1.27, 95%CI:1.10–1.47) were all not reached the significance threshold of 5.05E-04 (Table 2; Figure 1B). Our data indicated that there existed genetic heterogeneity among BCLO, UCLO, RCLO, and LCLO.

**TABLE 2:** Replication of the association analysis in 1p22 among sub-phenotype of NSCLO.

SNP	A1	BCLO		LCLO		RCLO		UCLO	
		*P*	OR (95%CI)	*P*	OR (95%CI)	*P*	OR (95%CI)	*P*	OR (95%CI)
rs2282229	A	0.026	0.71 (0.52–0.96)	0.031	0.72 (0.54–0.97)	0.038	0.73 (0.54–0.98)	0.039	0.74 (0.55–0.99)
rs11165065	A	0.046	0.77 (0.59–1.00)	0.065	0.79 (0.62–1.02)	0.056	0.78 (0.60–1.01)	0.067	0.79 (0.62–1.02)
rs2297636	C	0.019	1.19 (1.03–1.37)	0.034	1.16 (1.01–1.33)	0.039	1.16 (1.01–1.33)	0.044	1.15 (1.00–1.31)
rs10782976	G	0.055	0.86 (0.74–1.00)	0.028	0.85 (0.73–0.98)	0.068	0.87 (0.75–1.01)	0.039	0.86 (0.74–0.99)
rs4147804	A	0.067	0.87 (0.74–1.01)	0.039	0.86 (0.74–0.99)	0.087	0.88 (0.75–1.02)	0.054	0.87 (0.75–1.00)
rs4147803	C	0.053	0.86 (0.74–1.00)	0.030	0.85 (0.73–0.98)	0.070	0.87 (0.75–1.01)	0.043	0.86 (0.75–1.00)
rs3761911	A	0.079	0.87 (0.75–1.02)	0.040	0.86 (0.74–0.99)	0.114	0.89 (0.76–1.03)	0.063	0.87 (0.76–1.01)
rs1931572	C	0.080	0.87 (0.75–1.02)	0.041	0.86 (0.74–0.99)	0.115	0.89 (0.76–1.03)	0.064	0.87 (0.76–1.01)
rs12407620	A	0.079	0.87 (0.75–1.02)	0.040	0.86 (0.74–0.99)	0.114	0.89 (0.76–1.03)	0.063	0.87 (0.76–1.01)
rs1931571	T	0.080	0.87 (0.75–1.02)	0.041	0.86 (0.74–0.99)	0.115	0.89 (0.76–1.03)	0.064	0.87 (0.76–1.01)
rs12730118	A	0.065	0.87 (0.74–1.01)	0.033	0.85 (0.74–0.99)	0.095	0.88 (0.76–1.02)	0.052	0.87 (0.75–1.00)
rs7550646	G	0.065	0.87 (0.74–1.01)	0.033	0.85 (0.74–0.99)	0.095	0.88 (0.76–1.02)	0.053	0.87 (0.75–1.00)
rs6698524	G	0.065	0.87 (0.74–1.01)	0.033	0.85 (0.74–0.99)	0.095	0.88 (0.76–1.02)	0.053	0.87 (0.75–1.00)
rs6701591	A	0.066	0.87 (0.74–1.01)	0.033	0.85 (0.74–0.99)	0.096	0.88 (0.76–1.02)	0.053	0.87 (0.75–1.00)
rs34497591	T	0.065	0.87 (0.74–1.01)	0.033	0.85 (0.74–0.99)	0.095	0.88 (0.76–1.02)	0.053	0.87 (0.75–1.00)
rs1931569	A	0.065	0.87 (0.74–1.01)	0.033	0.85 (0.74–0.99)	0.095	0.88 (0.76–1.02)	0.053	0.87 (0.75–1.00)
rs1931568	G	0.065	0.87 (0.74–1.01)	0.033	0.85 (0.74–0.99)	0.095	0.88 (0.76–1.02)	0.053	0.87 (0.75–1.00)
rs1931567	C	0.065	0.87 (0.74–1.01)	0.033	0.85 (0.74–0.99)	0.095	0.88 (0.76–1.02)	0.053	0.87 (0.75–1.00)
rs34781620	G	0.065	0.87 (0.74–1.01)	0.033	0.85 (0.74–0.99)	0.095	0.88 (0.76–1.02)	0.053	0.87 (0.75–1.00)
**rs12071152**	**A**	0.002	1.28 (1.10–1.49)	**4.00E-04**	**1.30(1.12–1.51)**	0.005	1.24 (1.07–1.45)	0.001	1.27 (1.10–1.47)
rs17398522	C	0.065	0.87 (0.74–1.01)	0.033	0.85 (0.74–0.99)	0.095	0.88 (0.76–1.02)	0.053	0.87 (0.75–1.00)
rs6686599	A	0.055	0.86 (0.74–1.00)	0.034	0.85 (0.74–0.99)	0.083	0.88 (0.75–1.02)	0.054	0.87 (0.75–1.00)
rs7546201	A	0.030	0.84 (0.72–0.98)	0.015	0.83 (0.72–0.97)	0.048	0.86 (0.74–1.00)	0.026	0.85 (0.74–0.98)
rs6541410	G	0.034	0.85 (0.73–0.99)	0.017	0.84 (0.72–0.97)	0.053	0.86 (0.74–1.00)	0.029	0.85 (0.74–0.98)
rs58544825	A	0.042	0.85 (0.73–0.99)	0.021	0.84 (0.73–0.97)	0.064	0.87 (0.75–1.01)	0.035	0.86 (0.74–0.99)
rs7512276	G	0.047	0.86 (0.73–1.00)	0.023	0.84 (0.73–0.98)	0.070	0.87 (0.75–1.01)	0.037	0.86 (0.74–0.99)
rs2483793	A	0.046	0.86 (0.73–1.00)	0.021	0.84 (0.73–0.97)	0.068	0.87 (0.75–1.01)	0.035	0.86 (0.74–0.99)
rs7551877	A	0.072	0.87 (0.74–1.01)	0.046	0.86 (0.74–1.00)	0.091	0.88 (0.75–1.02)	0.064	0.87 (0.75–1.01)

The table shows SNPs with *p* < 0.05 in the replication phase. A1, minor allele; SNP, single nucleotide polymorphism; BCLO, bilateral cleft lip only; UCLO, unilateral cleft lip only; RCLO, right cleft lip only; LCLO, left cleft lip only; OR refers to odds ratio. 95%CI refers to 95% confidence interval. P refers to *P*-value for this test. The bold characters indicated the significant SNPs after multiple corrections (significant threshold is 5.05E-04).

### De novo harmful rare variant *ARHGAP29* ^*R551T*^ was identified to be associated with NSCLP

Three harmful rare variants were identified to be novel (NM_000350.2: c.979C>T in *ABCA4* and NM_004815.3: c.1652G>C and NM_004815.3: c.559G>A in *ARHGAP29*), which have not been reported in public databases such as 1000 Genome, Esp6500, ExAC, and GnomAD.

**FIGURE 2:**
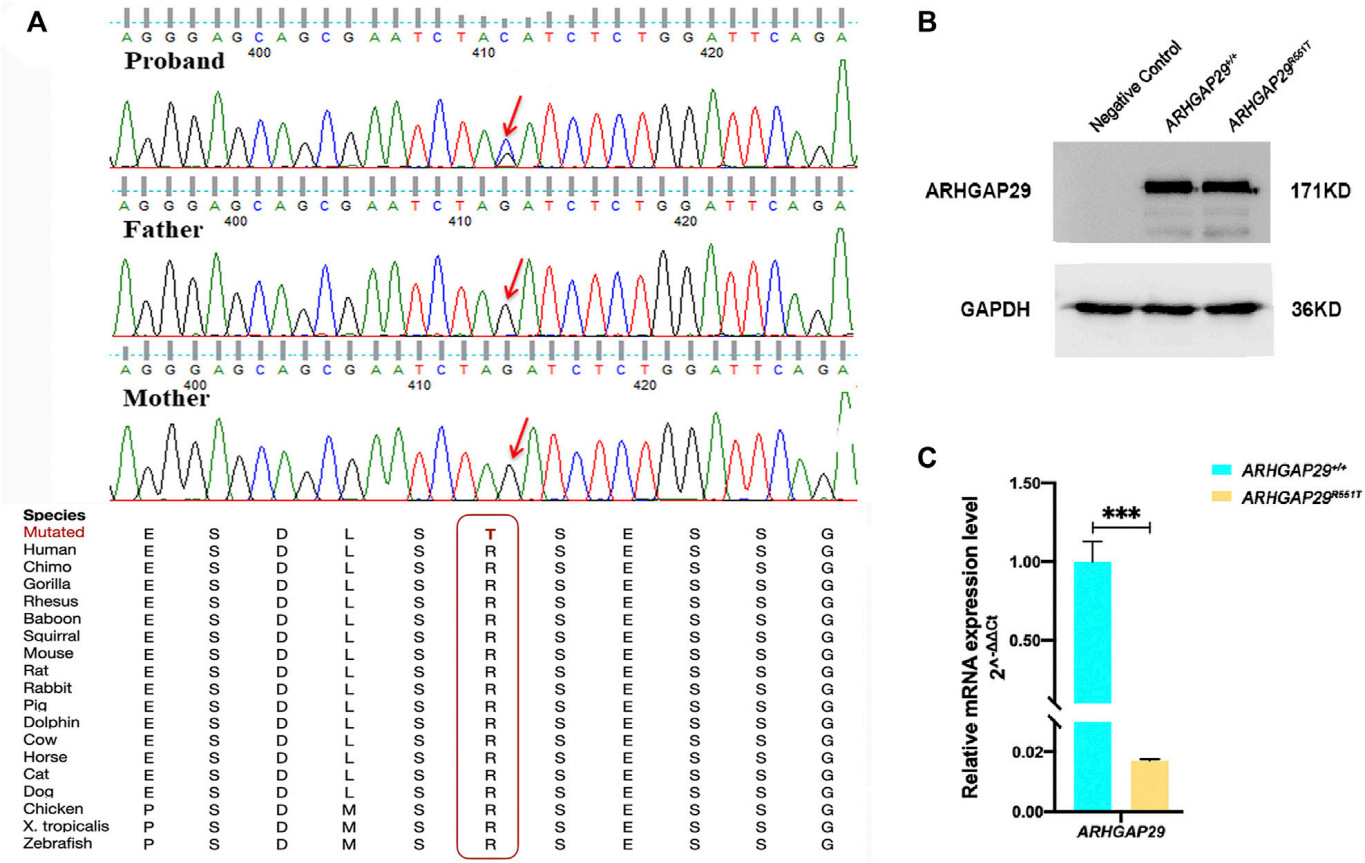
**(A)** Sanger sequencing results of the *de novo* harmful rare variant in *ARHGAP29*. Sequence chromatograms indicate the heterozygous variant (NM_004815.3, NP_004806.3; c.1652G>C, p.R551T). The red letter and box emphasize the cross-species conservation of the altered amino acid. **(B, C)** Western blot and RT-qPCR analysis of the *ARHGAP29* expression in HEK-293T cells 48 h after plasmid transfection. The results are presented as mean values with standard deviation (SD) normalized to *GAPDH*, and there were three biological replicates, ***p < 0.001.

We validated all of them by Sanger sequencing on carriers and their parents, through which NM_000350.2: c.979C>T in *ABCA4* and NM_004815.3: c.559G>A in *ARHGAP29* were shown to be inherited from the parents of carriers, whereas NM_004815.3: c.1652G>C in *ARHGAP29* was proved to be *de novo*, and it resulted in a missense mutation of 551 amino acids (p.R551T) of ARHGAP29 that is highly conserved across several species (Figure 2A). Then, NM_004815.3: c.1652G>C was further screened among 508 NSCLP cases and 438 normal controls, but it did not appear. Based on the conservation and harmfulness, we speculated that NM_004815.3: c.1652G>C in *ARHGAP29*, a *de novo* harmful rare variant, would be a risk factor for NSCLP.

### *ARHGAP29* ^*R551T*^ results in a decreased expression level of *ARHGAP29 in vitro*

Expression of fluorescence demonstrated that both pcDNA3.1-*ARHGAP29* and pcDNA3.1-*ARHGAP29* ^*R551T*^ were efficiently expressed in HEK-293T cells. Western Blot revealed that the expression levels of ARHGAP29 in homozygous *Arhgap29* ^*R553T/R553T*^ and wild-type group were comparable (Figure 2B). However, compared to the wild-type group, RT-qPCR revealed that homozygous *Arhgap29* ^*R553T/R553T*^ led to the lower mRNA expression level of *ARHGAP29* (Figure 2C).

**FIGURE 3:**
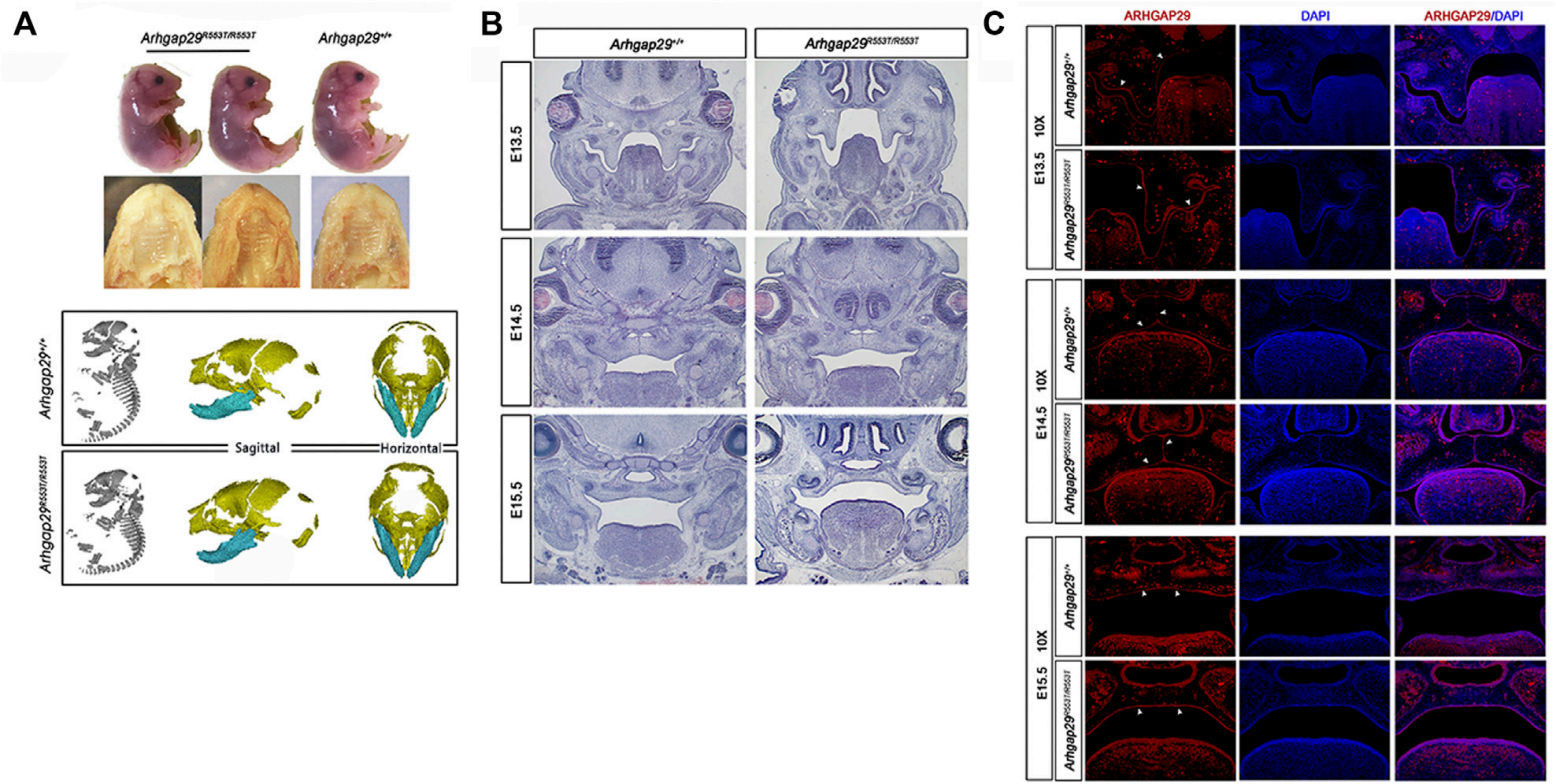
**(A)**. Macroscopic and palatal phenotypes of E18.5 embryos; (**B,C)** HE and immunofluorescence staining of palatal coronal sections of E13.5–E15.5 embryos.

Additionally, we examined its effect *in vivo*. At E18.5, there were no significant differences in body length, craniofacial morphology, or bone growth between *Arhgap29* ^*R553T/R553T*^ and wild-type mice embryos, and no overt cleft palate phenotype was observed (Figure 3A). From E13.5 to E15.5, HE images of coronal sections showed normal elevation and fusion of palate shelves in both *Arhgap29* ^*R553T/R553T*^ and wild-type mice embryos (Figure 3B). Furthermore, ARHGAP29 was expressed similarly in the palatal epithelium of *Arhgap29* ^*R553T/R553T*^ and wild-type mice embryos (Figure 3C).

### *ARHGAP29* ^*R551T*^ affects NSCL/P-related biological processes

Even though considering the decreased expression mRNA level of *ARHGAP29 in vitro*, we decided to further explore the influence of *Arhgap29* ^*R553T/R553T*^ on the transcriptome *in vivo* by RNA sequencing on the secondary palate tissue of E13.5 homozygous *Arhgap29* ^*R553T/R553T*^ and wild-type mice embryos. As predicted, the expression level of the *Arhgap29* gene transcript NM_172525.2, which is identical to the transcript, where the *de novo* harmful rare variant NM_004815.3: c.1652G>C located at the human genome, was also significantly downregulated when compared to the expression level in wild-type mice.

**FIGURE 4:**
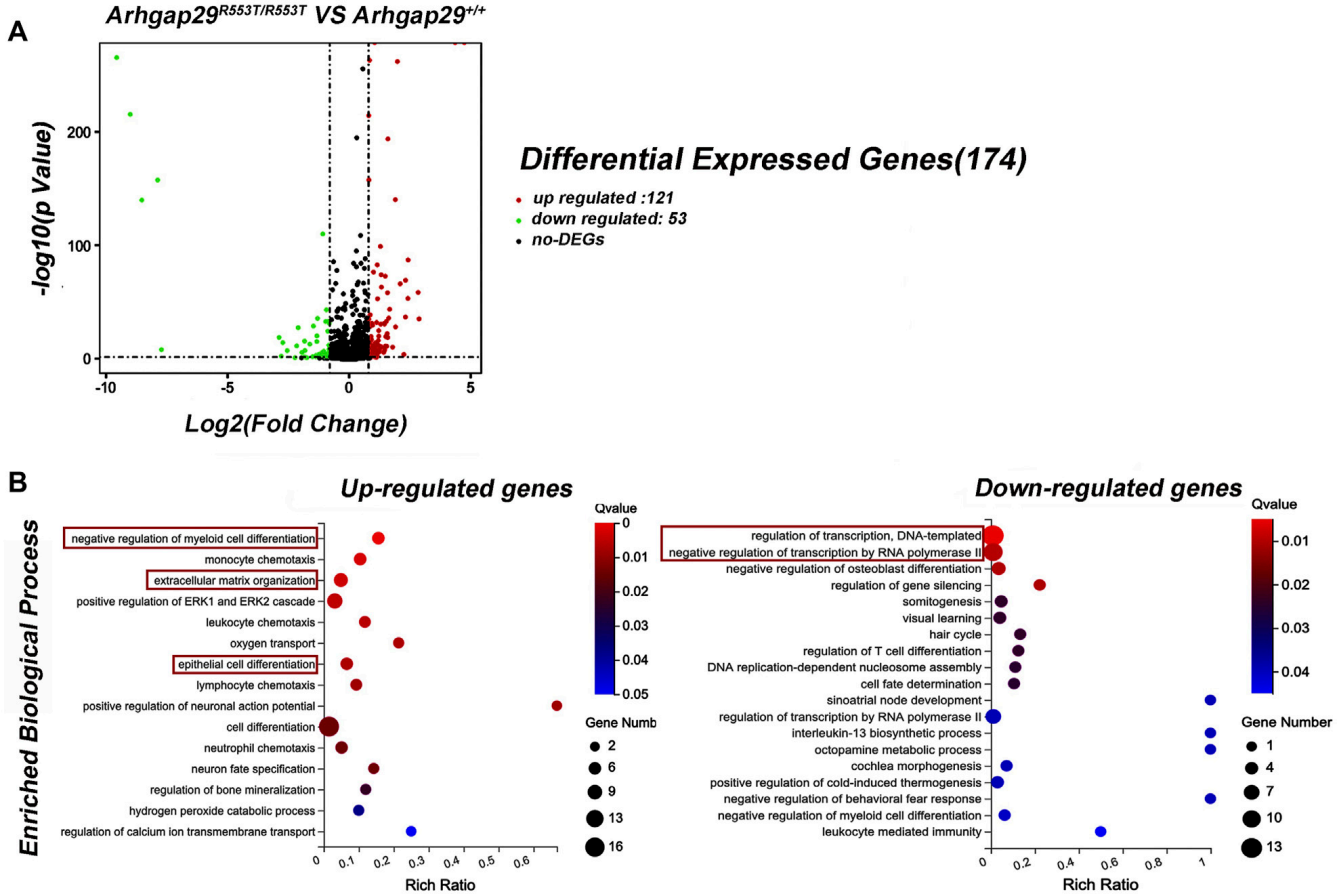
Results of RNA sequencing on *Arhgap29* ^R553T/R553T^ and wild-type mice. **(A)** Volcanic maps of differential expression genes. **(B)** GO analysis of DEGs. All the shown GO terms were significantly enriched with Q-value less than 0.05.

In addition, decreased *Arhgap29* led to significant changes in 174 genes, 121 of which were upregulated and 53 of which were downregulated (Figure 4A). The conditions for differential gene expression analysis include FPKM (wild-type)> 1, |log2|≥ 0.8, and *p* < 0.05.

Gene Ontology (GO) analysis for DEGs revealed that 15 biological processes were significantly enriched in upregulated genes, of which “epithelial cell differentiation” was the most relevant term to NSCL/P. In addition, most downregulated genes were significantly enriched in biological processes related to transcription, such as “regulation of transcription, DNA-templated” and “negative regulation of transcription by RNA polymerase II” (Figure 4B).

## Discussion

Since GWAS indicated that 1p22 was associated with NSCL/P (Beaty et al., 2010), a large number of common and rare variants have been identified in this region (Leslie et al., 2012; Butali et al., 2014; Chandrasekharan and Ramanathan, 2014; Letra et al., 2014; Gowans et al., 2016; Savastano et al., 2017). In this study, we aim to identify additional susceptibility variants for NSCL/P in the 1p22 region among the Han Chinese population using deep sequencing.

For common variants, we performed an initial association analysis and additional replications to investigate their associations. We found that rs77179923 was specifically associated with NSCLP (*p* = 4.70E-04, OR = 1.84, 95%CI: 1.31–2.58) (Table 1); rs77179923 is located in the introns of *ABCA4* gene, it was once reported to be significantly associated with NSCL/P among Asian trios by Leslie et al. (2015), but subsequent research indicated that it may not be functional (Liu et al., 2017a). In addition, rs12071152 was marginally associated with NSCLO (*p* = 9.40E-04, OR = 1.27, 95%CI: 1.10–1.46) (Table 1), and we first identified its unique association with LCLO (*p* = 4.00E-04, OR = 1.30, 95%CI: 1.12–1.51) (Table 2); since it is located in the intergenic non-coding region, we used HaploReg (Version v4.1) and RegulomeDB (Version 2.0.3) to annotate rs12071152, and its A allele was observed to have altered seven motifs and showed four eQTL signals (Supplementary Tables S6, S7, and Supplementary Figure S2); these results suggested that rs12071152 is a regulatory SNP, it may function by affecting the expression of ARHGAP29 in the etiology of LCLO, whereas further validation is required.

So far, we have identified two SNPs that are specifically associated with NSCLP or LCLO. However, none were associated with the other sub-phenotypes of NSCLO, indicating genetic heterogeneity among sub-phenotypes of NSCLO. In fact, numerous studies support this viewpoint. Carlson et al. (2017) found that different regions on chromosome 13 were specifically associated with UCLO and BCLO among Asian populations; our previous study also indicated that rs1345186 in the *TP63* gene was significantly associated with RCLO rather than LCLO and BCLO among the Han Chinese population (Yin et al., 2021). Moreover, these results remind us that the risk variants are likely to be masked by other signals when the association analysis was performed between controls and cases containing several subtypes. Consequently, a more detailed classification of the phenotype will be required in future genetic research to unearth more susceptibility variants.

Rare variants, with MAF less than 0.05% (Wagner, 2013), have a higher contribution to complex traits, meaning they confer a larger effect size than common variants; a portion of the common variants that show significant association with diseases are likely to be driven by rare variant (Bodmer and Bonilla, 2008; Nelson et al., 2012; Tennessen et al., 2012; Tada et al., 2016). So far, it has been reported that rare variants of *ARHGAP29* play crucial roles in the etiology of NSCL/P (Chandrasekharan and Ramanathan, 2014; Liu et al., 2017b; Paul et al., 2017; Savastano et al., 2017). Liu et al. (2017b) once identified a rare variant (NM_004815.3, NP_004806.3; c.1654T>C, p.Ser552Pro) adjacent to ours in a European CPO-defected family. This mutation decreased the stability of ARHGAP29, which inhibits cell migration in immortalized human keratinocytes (iNHKs); the mutant zebrafish failed to delay epiboly, and it was thus suggested to be a loss-of-function variant (Liu et al., 2017b). In addition, Paul et al. (2017) identified a point mutation p.*K326X* at *ARHGAP29* in the NSCL/P case, and the heterozygous *Arhgap29* ^*K326X*^ mutant mouse had abnormal adhesion prior to the formation of the palate.

Here, we identified a heterozygous missense variant NM_004815.3, NP_004806.3; c.1652G>C, p.R551T (*ARHGAP29*) that was *de novo*, highly conserved across species; it is predicted to be harmful by all *in silico* tools, and it is a pathogenic variant by the ACMG guideline (PS2, PS3, and PM2) (Richards et al., 2015). Based on this evidence and its adjacent missense mutation p.Ser552Pro functions as a loss-of-function variant (Liu et al., 2017b), we constructed a mouse model harboring *Arhgap29* ^*R553T*^, which is identical to *ARHGAP29* ^*R551T*^, to clarify its function. However, neither overt cleft palate phenotype nor abnormal palate shelves elevation or fusion was observed (Figure 3A, B). We inferred that this attributed to the insufficient dose effect of *Arhgap29* ^*R553T/R553T*^, because Western blot and immunofluorescence assay demonstrated that both *ARHGAP29* ^*R551T*^ and *Arhgap29* ^*R553T*^ did not affect the expression of ARHGAP29 protein (Figure 2B and Figure 3C). In addition, NSCL/P is a polygenic disease involving multiple genes; thus, the effect of *ARHGAP29* ^*R551T*^ may be compensated by other genes or its functions in the etiology of NSCL/P by interacting with other genes in the signaling cascade of craniofacial embryology. Even though we cannot ignore the change of mRNA expression level of *ARHGAP29* result from *ARHGAP29* ^*R551T*^, this mouse model is valuable for identifying covariates of *Arhgap29* ^*R553T/R553T*^ at the transcriptome level, as well as discovering more NSCL/P-associated biological processes that *ARHGAP29* might participate in (Paul et al., 2017). In this study, we found that the genes affected by *Arhgap29* ^*R553T/R553T*^ were enriched in the biological processes of epithelial cell differentiation and transcriptional regulation that may be related to NSCL/P, but whether they are truly involved in the occurrence of NSCL/P needs further in-depth research.

In conclusion, via targeted sequencing on 1p22 among the Han Chinese population, we found that rs77179923 was specifically associated with NSCLP; rs12071152 was significantly and specifically associated with LCLO. In addition, *de novo* harmful rare variants NM_004815.3, NP_004806.3; c.1652G>C, p.R551T (ARHGAP29), which decreased *ARHGAP29* expression, were identified to be a risk factor for NSCLP. We generated a mouse model harboring variant identical to the *de novo* harmful variants; although no overt phenotype was observed, several susceptibility NSCL/P-related biological processes that are affected by *Arhgap29* ^*R553T/R553T*^ were observed after RNA-sequencing of the E13.5 secondary palate; however, the mechanism requires further investigation.

## Data Availability

All the datasets generated in this article were shown in the main text and the Supplementary Material. Any questions about the data, please contact to zhonglinjia@sina.com.
