# Supplementary material for: Targeted re-sequencing on 1p22 among non-syndromic orofacial clefts from Han Chinese population
Source: Front Genet. 2022 Aug 17;13:947126. doi: 10.3389/fgene.2022.947126 (PMC9428125; doi:10.3389/fgene.2022.947126)
Supplement: Supplementary file 1 [file DataSheet1.docx]

**Targeted re-sequencing on 1p22 among non-syndromic orofacial clefts from Han Chinese population**

Mu-Jia Li ^1,2^, Jia-Yu Shi^3^, Bi-He Zhang^1,2^, Qian-Ming Chen^1^, Bing Shi^1,2^, Zhong-Lin Jia^1,2,*^

| Supplementary Table 1 Details of samples used in the study | | | | | | | | | |
| --- | --- | --- | --- | --- | --- | --- | --- | --- | --- |
|  | | | | Total | Bilateral | Unilateral | Unilateral right | Unilateral left | Controls |
| **Deep sequencing phase** | | NSCL/P | | 159 | 25 | 109 | 48 | 61 | 542* |
|  |  | NSCLP | | 80 | 15 | 46 | 21 | 25 |  |
|  |  | NSCLO | | 79 | 10 | 63 | 27 | 36 |  |
| **Replication phase** | Association analysis^#^ | NSCL/P | | 1626 | 201 | 1047 | 377 | 670 | 2255 |
|  |  | NSCLP | | 579 | 103 | 236 | 85 | 151 |  |
|  |  | NSCLO | | 1047 | 98 | 811 | 292 | 519 |  |
|  | Sanger sequencing | | NSCLO | 508 | 508 | / | / | / | 438 |
| Note：NSCL/P, non-syndromic cleft lip with or without palate; NSCLP, non-syndromic cleft lip with cleft palate; NSCLO, non-syndromic cleft lip only; UCLO, unilateral cleft lip only; LCLO, left cleft lip only; RCLO, right cleft lip only; BCLO, bilateral cleft lip only; *, this part of controls’ data come from Novogene internal database (http://www.novogene.com/); ^#^, these genotyping data of Han Chinese population come from two GWAS (PMID: 25775280, PMID: 31609978 ) . | | | | | | | | | |

| **Supplementary Table 2** Primers of the novel variants | | | | | |
| --- | --- | --- | --- | --- | --- |
| Variations | Gene | Primers (5' to 3') | | PCR Products | Sanger Validation |
| NM_000350.2:c.979C>T | *ABCA4* | F | CTCAGCAAGACAAGACAGATGC | 492 bp | Novel |
|  |  | R | GCTGGATTAAGGATTGCTGAGA |  |  |
| NM_004815.3:c.1652G>C | *ARHGAP29* | F | TGAGTTTTCATTGCCAGACTTAAA | 507 bp | De novo |
|  |  | R | GACCTGCCAACTCTTTAGAGGA |  |  |
| NM_004815.3:c.559G>A | *ARHGAP29* | F | ACTCCAAACAAAGAAGCACTGA | 520 bp | Novel |
|  |  | R | AGAGTAACAAATTGGGGCAAAA |  |  |

| **Supplementary Table 3** Primers of the RT-qPCR | | |
| --- | --- | --- |
| Gene | Primers (5' to 3') | |
| *ARHGAP29* | F | GACTTTCATCGAAAACTTCCACG |
|  | R | AATTTGCGAAACTTGTGTGTGAG |
| *GAPDH* | F | TGACTTCAACAGCGACACCCA |
|  | R | CACCCTGTTGCTGTAGCCAAA |

| **Supplementary Table 4** Association analysis of common variants in the discovery phase | | | | | | | | |
| --- | --- | --- | --- | --- | --- | --- | --- | --- |
| SNP | Chr | Position  (Hg19) | Alt/Ref Allele | MAF | | P_HWE_ | P | OR (95%CI) |
|  |  |  |  | NSCL/P | Control |  |  |  |
| rs4147803 | 1 | 94582293 | C/G | 0.24 | 0.36 | 0.93 | 3.42E-05 | 0.55(0.73,0.41) |
| rs10782976 | 1 | 94581125 | A/G | 0.25 | 0.37 | 0.85 | 5.12E-05 | 1.78(2.36,1.34) |
| rs7551877 | 1 | 94606077 | A/G | 0.23 | 0.35 | 0.40 | 5.49E-05 | 0.56(0.75,0.42) |
| rs4147804 | 1 | 94582227 | A/G | 0.24 | 0.36 | 0.93 | 6.18E-05 | 0.56(0.75,0.42) |
| rs2483793 | 1 | 94605494 | C/A | 0.24 | 0.36 | 0.64 | 8.05E-05 | 1.76(2.34,1.32) |
| rs6686599 | 1 | 94596831 | A/G | 0.24 | 0.36 | 0.78 | 1.09E-04 | 0.57(0.76,0.43) |
| rs1931572 | 1 | 94588992 | T/C | 0.24 | 0.36 | 0.71 | 1.41E-04 | 1.73(2.30,1.30) |
| rs12407620 | 1 | 94589213 | A/C | 0.24 | 0.36 | 0.71 | 1.41E-04 | 0.58(0.77,0.43) |
| rs1931571 | 1 | 94589249 | A/T | 0.24 | 0.36 | 0.71 | 1.41E-04 | 1.73(2.30,1.30) |
| rs7550646 | 1 | 94590532 | G/A | 0.24 | 0.36 | 0.71 | 1.41E-04 | 0.58(0.77,0.43) |
| rs6698524 | 1 | 94590962 | G/A | 0.24 | 0.36 | 0.71 | 1.41E-04 | 0.58(0.77,0.43) |
| rs6701591 | 1 | 94590970 | A/T | 0.24 | 0.36 | 0.71 | 1.41E-04 | 0.58(0.77,0.43) |
| rs34497591 | 1 | 94591795 | T/C | 0.24 | 0.36 | 0.71 | 1.41E-04 | 0.58(0.77,0.43) |
| rs1931569 | 1 | 94592762 | A/G | 0.24 | 0.36 | 0.71 | 1.41E-04 | 0.58(0.77,0.43) |
| rs1931568 | 1 | 94592839 | G/A | 0.24 | 0.36 | 0.71 | 1.41E-04 | 0.58(0.77,0.43) |
| rs1931567 | 1 | 94592964 | C/T | 0.24 | 0.36 | 0.71 | 1.41E-04 | 0.58(0.77,0.43) |
| rs34781620 | 1 | 94593069 | G/A | 0.24 | 0.36 | 0.71 | 1.41E-04 | 0.58(0.77,0.43) |
| rs3761911 | 1 | 94588754 | A/T | 0.24 | 0.36 | 0.78 | 1.42E-04 | 0.58(0.77,0.44) |
| rs17398522 | 1 | 94594521 | C/A | 0.24 | 0.36 | 0.78 | 1.42E-04 | 0.58(0.77,0.44) |
| rs6541410 | 1 | 94600206 | C/G | 0.25 | 0.36 | 1.00 | 1.52E-04 | 1.72(2.28,1.30) |
| rs7512276 | 1 | 94604714 | G/C | 0.25 | 0.36 | 1.00 | 1.52E-04 | 0.58(0.77,0.44) |
| rs1191228 | 1 | 94532562 | C/T | 0.29 | 0.19 | 0.39 | 1.56E-04 | 0.57(0.76,0.43) |
| rs12730118 | 1 | 94589913 | A/T | 0.24 | 0.35 | 0.57 | 1.82E-04 | 0.58(0.77,0.44) |
| rs58544825 | 1 | 94602571 | A/G | 0.25 | 0.36 | 0.85 | 1.96E-04 | 0.59(0.78,0.44) |
| rs2151846 | 1 | 94587687 | G/T | 0.40 | 0.48 | 0.61 | 4.41E-04 | 0.63(0.82,0.49) |
| rs7546201 | 1 | 94598783 | A/G | 0.25 | 0.36 | 0.93 | 5.05E-04 | 0.61(0.80,0.46) |
| rs61245991 | 1 | 94573636 | A/G | 0.28 | 0.39 | 0.53 | 5.60E-04 | 0.62(0.81,0.47) |
| rs481931 | 1 | 94570016 | T/G | 0.28 | 0.39 | 0.59 | 7.11E-04 | 0.63(0.82,0.48) |
| rs4147811 | 1 | 94575056 | T/C | 0.29 | 0.39 | 0.42 | 7.25E-04 | 0.63(0.82,0.48) |
| rs3827712 | 1 | 94575171 | C/T | 0.29 | 0.39 | 0.42 | 7.25E-04 | 0.63(0.82,0.48) |
| rs3789451 | 1 | 94586328 | T/C | 0.29 | 0.39 | 0.59 | 7.25E-04 | 0.63(0.82,0.48) |
| rs4147816 | 1 | 94574780 | T/C | 0.29 | 0.39 | 0.53 | 7.32E-04 | 0.63(0.83,0.48) |
| rs4147812 | 1 | 94575043 | C/A | 0.29 | 0.39 | 0.42 | 7.32E-04 | 0.63(0.83,0.48) |
| rs3789432 | 1 | 94575308 | C/T | 0.29 | 0.39 | 0.42 | 7.32E-04 | 0.63(0.83,0.48) |
| rs12121974 | 1 | 94454018 | A/G | 0.43 | 0.33 | 0.10 | 8.57E-04 | 1.55(2.00,1.20) |
| rs3789431 | 1 | 94573046 | T/C | 0.29 | 0.39 | 0.47 | 9.11E-04 | 0.63(0.83,0.48) |
| rs11165083 | 1 | 94596291 | G/A | 0.30 | 0.40 | 0.59 | 1.58E-03 | 0.65(0.85,0.50) |
| rs581244 | 1 | 94533990 | T/A | 0.27 | 0.18 | 0.57 | 1.94E-03 | 0.62(0.83,0.46) |
| rs17394432 | 1 | 94594848 | G/A | 0.30 | 0.40 | 0.65 | 1.95E-03 | 0.65(0.85,0.50) |
| rs11802196 | 1 | 94594043 | C/A | 0.30 | 0.40 | 0.65 | 1.96E-03 | 0.66(0.86,0.50) |
| rs11165081 | 1 | 94594080 | C/A | 0.30 | 0.40 | 0.65 | 1.96E-03 | 0.66(0.86,0.50) |
| rs12070274 | 1 | 94466090 | T/A | 0.05 | 0.02 | 1.00 | 3.40E-03 | 2.90(5.71,1.47) |
| rs7413585 | 1 | 94606871 | C/T | 0.25 | 0.17 | 0.88 | 3.43E-03 | 1.57(2.12,1.17) |
| rs3789452 | 1 | 94587501 | A/T | 0.20 | 0.28 | 0.92 | 3.64E-03 | 0.64(0.86,0.47) |
| rs4434844 | 1 | 94602475 | A/C | 0.31 | 0.40 | 0.25 | 3.87E-03 | 1.48(1.93,1.13) |
| rs570926 | 1 | 94570218 | C/T | 0.34 | 0.43 | 0.43 | 5.30E-03 | 0.69(0.89,0.53) |
| rs2297636 | 1 | 94568822 | C/T | 0.50 | 0.41 | 0.22 | 5.63E-03 | 1.43(1.84,1.11) |
| rs560426 | 1 | 94553438 | T/C | 0.38 | 0.29 | 0.84 | 5.91E-03 | 0.69(0.89,0.53) |
| rs1209515 | 1 | 94571335 | C/T | 0.36 | 0.44 | 0.60 | 8.17E-03 | 0.71(0.91,0.54) |
| rs77865902 | 1 | 94718355 | A/C | 0.07 | 0.03 | 0.47 | 1.02E-02 | 2.10(3.62,1.22) |
| rs12070273 | 1 | 94466088 | G/A | 0.05 | 0.02 | 1.00 | 1.20E-02 | 2.34(4.46,1.23) |
| rs11583428 | 1 | 94597552 | T/G | 0.20 | 0.14 | 0.37 | 1.26E-02 | 1.54(2.13,1.11) |
| rs11588391 | 1 | 94599871 | T/C | 0.24 | 0.18 | 0.55 | 1.47E-02 | 1.47(1.98,1.09) |
| rs11165079 | 1 | 94589761 | T/G | 0.24 | 0.18 | 0.56 | 1.50E-02 | 1.46(1.97,1.08) |
| rs3789445 | 1 | 94582249 | G/T | 0.41 | 0.33 | 0.44 | 1.61E-02 | 1.37(1.78,1.06) |
| rs11165088 | 1 | 94621913 | C/T | 0.14 | 0.10 | 0.20 | 1.78E-02 | 1.58(2.29,1.09) |
| rs61784009 | 1 | 94598281 | C/A | 0.24 | 0.18 | 0.38 | 1.87E-02 | 1.45(1.96,1.07) |
| rs1931566 | 1 | 94593100 | G/C | 0.24 | 0.18 | 0.66 | 1.90E-02 | 1.44(1.95,1.07) |
| rs4147798 | 1 | 94585009 | T/C | 0.23 | 0.17 | 0.55 | 2.14E-02 | 1.44(1.95,1.06) |
| rs570878 | 1 | 94570234 | T/G | 0.39 | 0.32 | 0.49 | 2.20E-02 | 0.74(0.95,0.57) |
| rs3789440 | 1 | 94579672 | A/G | 0.23 | 0.17 | 0.46 | 2.21E-02 | 1.44(1.95,1.06) |
| rs12025221 | 1 | 94580059 | C/T | 0.23 | 0.17 | 0.46 | 2.21E-02 | 1.44(1.95,1.06) |
| rs74102058 | 1 | 94468293 | C/T | 0.05 | 0.02 | 1.00 | 2.32E-02 | 2.15(4.07,1.14) |
| rs11165080 | 1 | 94589849 | G/A | 0.24 | 0.18 | 0.66 | 2.35E-02 | 1.43(1.93,1.06) |
| rs1931570 | 1 | 94590549 | T/G | 0.24 | 0.18 | 0.66 | 2.35E-02 | 1.43(1.93,1.06) |
| rs2184451 | 1 | 94570625 | A/G | 0.39 | 0.32 | 0.49 | 2.61E-02 | 1.35(1.74,1.04) |
| rs3789421 | 1 | 94565577 | A/G | 0.23 | 0.17 | 0.55 | 2.65E-02 | 1.43(1.94,1.05) |
| rs3789439 | 1 | 94579426 | C/T | 0.23 | 0.18 | 0.30 | 2.74E-02 | 1.43(1.93,1.05) |
| rs12035820 | 1 | 94580233 | T/C | 0.23 | 0.18 | 0.46 | 2.74E-02 | 1.43(1.93,1.05) |
| rs3789442 | 1 | 94581456 | C/G | 0.23 | 0.18 | 0.46 | 2.74E-02 | 1.43(1.93,1.05) |
| rs3789443 | 1 | 94581529 | G/A | 0.23 | 0.18 | 0.46 | 2.74E-02 | 1.43(1.93,1.05) |
| rs3789444 | 1 | 94581540 | T/C | 0.23 | 0.18 | 0.46 | 2.74E-02 | 1.43(1.93,1.05) |
| rs1931564 | 1 | 94597160 | A/G | 0.23 | 0.18 | 0.46 | 2.74E-02 | 1.43(1.93,1.05) |
| rs12057375 | 1 | 94573722 | T/G | 0.33 | 0.27 | 0.44 | 2.77E-02 | 1.36(1.78,1.04) |
| rs10493869 | 1 | 94580874 | C/T | 0.23 | 0.18 | 0.55 | 2.77E-02 | 1.42(1.92,1.05) |
| rs10493870 | 1 | 94580906 | T/C | 0.23 | 0.18 | 0.55 | 2.77E-02 | 1.42(1.92,1.05) |
| rs3827714 | 1 | 94583589 | A/G | 0.23 | 0.18 | 0.55 | 2.77E-02 | 1.42(1.92,1.05) |
| rs12093963 | 1 | 94573920 | G/A | 0.33 | 0.27 | 0.44 | 2.80E-02 | 1.36(1.78,1.04) |
| rs3789434 | 1 | 94575978 | C/T | 0.33 | 0.27 | 0.44 | 2.80E-02 | 1.36(1.78,1.04) |
| rs3789438 | 1 | 94577462 | T/G | 0.33 | 0.27 | 0.38 | 2.81E-02 | 1.36(1.78,1.04) |
| rs4147810 | 1 | 94576664 | G/A | 0.33 | 0.27 | 0.59 | 2.84E-02 | 1.36(1.77,1.04) |
| rs553608 | 1 | 94531013 | C/T | 0.17 | 0.12 | 0.41 | 2.84E-02 | 0.67(0.95,0.47) |
| rs1889404 | 1 | 94577423 | T/C | 0.22 | 0.17 | 0.21 | 2.98E-02 | 1.43(1.95,1.05) |
| rs12088309 | 1 | 94563916 | C/T | 0.47 | 0.46 | 1.00 | 2.98E-02 | 1.33(1.71,1.03) |
| rs7366102 | 1 | 94532262 | C/T | 0.16 | 0.11 | 0.83 | 3.29E-02 | 0.67(0.95,0.47) |
| rs4147801 | 1 | 94584290 | C/T | 0.23 | 0.17 | 0.46 | 3.32E-02 | 1.41(1.91,1.04) |
| rs2297635 | 1 | 94576893 | A/G | 0.33 | 0.27 | 0.38 | 3.33E-02 | 1.35(1.77,1.03) |
| rs11165076 | 1 | 94583698 | T/G | 0.23 | 0.18 | 0.46 | 3.36E-02 | 1.40(1.90,1.03) |
| rs1889406 | 1 | 94572890 | A/C | 0.35 | 0.28 | 0.67 | 3.66E-02 | 0.75(0.98,0.58) |
| rs1889405 | 1 | 94577410 | T/C | 0.22 | 0.17 | 0.28 | 3.77E-02 | 1.40(1.91,1.03) |
| rs1211213 | 1 | 94571420 | G/A | 0.23 | 0.29 | 1.00 | 3.86E-02 | 0.73(0.98,0.55) |
| rs11165065 | 1 | 94491468 | A/G | 0.09 | 0.06 | 0.25 | 3.98E-02 | 1.63(2.57,1.03) |
| rs2282229 | 1 | 94488326 | A/T | 0.07 | 0.04 | 1.00 | 4.10E-02 | 1.72(2.88,1.03) |
| rs4147807 | 1 | 94579053 | G/A | 0.23 | 0.18 | 0.19 | 4.27E-02 | 1.38(1.87,1.02) |
| rs77179923 | 1 | 94562770 | T/C | 0.08 | 0.05 | 0.19 | 4.49E-02 | 1.64(2.64,1.02) |
| rs140051427 | 1 | 94737309 | C/A | 0.05 | 0.03 | 1.00 | 4.55E-02 | 1.93(3.60,1.03) |
| rs12071152 | 1 | 94593399 | A/G | 0.36 | 0.30 | 0.61 | 4.66E-02 | 1.31(1.71,1.01) |
| rs1191232 | 1 | 94531192 | G/A | 0.17 | 0.12 | 0.31 | 4.74E-02 | 0.69(0.98,0.49) |
| rs3818778 | 1 | 94480529 | C/A | 0.20 | 0.16 | 0.07 | 4.89E-02 | 1.39(1.91,1.01) |
| **Note:** The table shows SNPs with P<0.05 in the discovery phase. SNP, single nucleotide polymorphism; Chr, chromosome; Alt/Ref，alternate/ reference allele; MAF, minor allele frequency; NSCL/P, non-syndromic cleft lip with or without palate; P_HWE_, P-value for Hardy-Weinberg equilibrium test; P, P-value for association analysis; OR, odds ratio; 95%CI, 95% confidence interval. | | | | | | | | |

| **Supplementary Table 5** Minor allele frequency and Hardy-Weinberg equilibrium test of the SNPs recruited in replication phase | | | | | | | | | | | | | | |
| --- | --- | --- | --- | --- | --- | --- | --- | --- | --- | --- | --- | --- | --- | --- |
| SNP | A1/A2  Allele | MAF | | | | | | | |  | HWE | | | |
|  |  | Control | NSCL/P | NSCLP | NSCLO | BCLO | LCLO | RCLO | UCLO |  | GENO | O(HET) | E(HET) | P_HWE_ |
| rs2282229 | A/T | 0.058 | 0.049 | 0.054 | 0.047 | 0.048 | 0.049 | 0.049 | 0.049 |  | 7/246/1995 | 0.11 | 0.11 | 1.00 |
| rs11165065 | A/G | 0.080 | 0.075 | 0.081 | 0.071 | 0.069 | 0.070 | 0.069 | 0.070 |  | 20/321/1907 | 0.14 | 0.15 | 0.12 |
| rs560426 | C/T | 0.320 | 0.336 | 0.325 | 0.343 | 0.344 | 0.344 | 0.343 | 0.343 |  | 232/973/1043 | 0.43 | 0.43 | 0.81 |
| rs77179923 | T/C | 0.032 | 0.029 | 0.055 | 0.015 | 0.007 | 0.007 | 0.008 | 0.008 |  | 2/138/2108 | 0.06 | 0.06 | 1.00 |
| rs12088309 | C/T | 0.453 | 0.468 | 0.467 | 0.469 | 0.469 | 0.467 | 0.467 | 0.467 |  | 464/1109/675 | 0.49 | 0.50 | 0.83 |
| rs2297636 | C/T | 0.415 | 0.432 | 0.423 | 0.438 | 0.437 | 0.435 | 0.435 | 0.434 |  | 403/1061/784 | 0.47 | 0.49 | 0.19 |
| rs12057375 | T/G | 0.279 | 0.296 | 0.306 | 0.291 | 0.293 | 0.293 | 0.291 | 0.292 |  | 177/902/1169 | 0.40 | 0.40 | 0.88 |
| rs3789434 | C/T | 0.280 | 0.297 | 0.305 | 0.292 | 0.294 | 0.294 | 0.293 | 0.293 |  | 177/904/1167 | 0.40 | 0.40 | 0.92 |
| rs4147810 | G/A | 0.281 | 0.297 | 0.307 | 0.292 | 0.293 | 0.294 | 0.292 | 0.293 |  | 180/902/1166 | 0.40 | 0.40 | 0.75 |
| rs2297635 | A/G | 0.278 | 0.295 | 0.302 | 0.290 | 0.293 | 0.294 | 0.291 | 0.292 |  | 175/900/1173 | 0.40 | 0.40 | 0.92 |
| rs3789438 | T/G | 0.279 | 0.297 | 0.304 | 0.292 | 0.294 | 0.295 | 0.293 | 0.294 |  | 175/905/1168 | 0.40 | 0.40 | 1.00 |
| rs10782976 | G/A | 0.326 | 0.310 | 0.315 | 0.306 | 0.297 | 0.295 | 0.298 | 0.296 |  | 241/982/1025 | 0.44 | 0.44 | 0.81 |
| rs4147804 | A/G | 0.320 | 0.305 | 0.310 | 0.302 | 0.293 | 0.291 | 0.294 | 0.292 |  | 232/976/1040 | 0.43 | 0.44 | 0.88 |
| rs4147803 | C/G | 0.323 | 0.305 | 0.308 | 0.304 | 0.294 | 0.293 | 0.296 | 0.294 |  | 239/973/1036 | 0.43 | 0.44 | 0.63 |
| rs3761911 | A/T | 0.327 | 0.309 | 0.313 | 0.307 | 0.296 | 0.295 | 0.298 | 0.296 |  | 245/978/1025 | 0.44 | 0.44 | 0.60 |
| rs1931572 | C/T | 0.327 | 0.309 | 0.313 | 0.307 | 0.297 | 0.295 | 0.299 | 0.297 |  | 245/979/1024 | 0.44 | 0.44 | 0.63 |
| rs12407620 | A/C | 0.327 | 0.309 | 0.313 | 0.307 | 0.296 | 0.295 | 0.298 | 0.296 |  | 246/977/1025 | 0.43 | 0.44 | 0.57 |
| rs1931571 | T/A | 0.327 | 0.309 | 0.313 | 0.307 | 0.297 | 0.295 | 0.299 | 0.297 |  | 246/978/1024 | 0.44 | 0.44 | 0.60 |
| rs11165079 | T/G | 0.205 | 0.199 | 0.190 | 0.204 | 0.211 | 0.206 | 0.213 | 0.208 |  | 99/722/1427 | 0.32 | 0.33 | 0.52 |
| rs11165080 | G/A | 0.205 | 0.199 | 0.190 | 0.204 | 0.211 | 0.206 | 0.213 | 0.208 |  | 99/724/1425 | 0.32 | 0.33 | 0.56 |
| rs12730118 | A/T | 0.327 | 0.309 | 0.313 | 0.307 | 0.296 | 0.295 | 0.298 | 0.296 |  | 246/977/1025 | 0.43 | 0.44 | 0.57 |
| rs7550646 | G/A | 0.326 | 0.308 | 0.310 | 0.306 | 0.296 | 0.295 | 0.298 | 0.296 |  | 245/974/1029 | 0.43 | 0.44 | 0.53 |
| rs1931570 | T/G | 0.205 | 0.199 | 0.191 | 0.204 | 0.211 | 0.206 | 0.213 | 0.208 |  | 100/723/1425 | 0.32 | 0.33 | 0.52 |
| rs6698524 | G/A | 0.326 | 0.308 | 0.311 | 0.306 | 0.296 | 0.295 | 0.298 | 0.296 |  | 246/974/1028 | 0.43 | 0.44 | 0.50 |
| rs6701591 | A/T | 0.326 | 0.308 | 0.311 | 0.306 | 0.297 | 0.295 | 0.299 | 0.297 |  | 246/975/1027 | 0.43 | 0.44 | 0.53 |
| rs34497591 | T/C | 0.326 | 0.308 | 0.311 | 0.306 | 0.296 | 0.295 | 0.298 | 0.296 |  | 246/975/1027 | 0.43 | 0.44 | 0.53 |
| rs1931569 | A/G | 0.326 | 0.308 | 0.310 | 0.306 | 0.296 | 0.295 | 0.298 | 0.296 |  | 245/975/1028 | 0.43 | 0.44 | 0.56 |
| rs1931568 | G/A | 0.326 | 0.308 | 0.310 | 0.306 | 0.296 | 0.295 | 0.298 | 0.296 |  | 245/975/1028 | 0.43 | 0.44 | 0.56 |
| rs1931567 | C/T | 0.326 | 0.307 | 0.310 | 0.305 | 0.296 | 0.295 | 0.298 | 0.296 |  | 245/974/1029 | 0.43 | 0.44 | 0.53 |
| rs34781620 | G/A | 0.326 | 0.308 | 0.310 | 0.306 | 0.296 | 0.295 | 0.298 | 0.296 |  | 245/975/1028 | 0.43 | 0.44 | 0.56 |
| rs1931566 | G/C | 0.205 | 0.199 | 0.190 | 0.203 | 0.210 | 0.205 | 0.212 | 0.207 |  | 100/721/1427 | 0.32 | 0.33 | 0.48 |
| rs12071152 | A/G | 0.308 | 0.338 | 0.340 | 0.337 | 0.338 | 0.342 | 0.336 | 0.340 |  | 219/948/1081 | 0.42 | 0.43 | 0.59 |
| rs17398522 | C/A | 0.326 | 0.308 | 0.310 | 0.306 | 0.296 | 0.295 | 0.298 | 0.296 |  | 245/975/1028 | 0.43 | 0.44 | 0.56 |
| rs6686599 | A/G | 0.326 | 0.307 | 0.309 | 0.305 | 0.295 | 0.294 | 0.297 | 0.296 |  | 245/976/1027 | 0.43 | 0.44 | 0.56 |
| rs7546201 | A/G | 0.329 | 0.309 | 0.314 | 0.307 | 0.297 | 0.295 | 0.299 | 0.297 |  | 249/982/1017 | 0.44 | 0.44 | 0.60 |
| rs6541410 | G/C | 0.329 | 0.309 | 0.314 | 0.307 | 0.297 | 0.295 | 0.299 | 0.297 |  | 249/982/1017 | 0.44 | 0.44 | 0.60 |
| rs58544825 | A/G | 0.328 | 0.308 | 0.314 | 0.305 | 0.296 | 0.295 | 0.298 | 0.296 |  | 249/978/1021 | 0.44 | 0.44 | 0.53 |
| rs7512276 | G/C | 0.332 | 0.311 | 0.316 | 0.307 | 0.299 | 0.297 | 0.300 | 0.299 |  | 252/989/1007 | 0.44 | 0.44 | 0.70 |
| rs2483793 | A/C | 0.333 | 0.311 | 0.316 | 0.308 | 0.299 | 0.297 | 0.300 | 0.299 |  | 252/994/1002 | 0.44 | 0.44 | 0.81 |
| rs7551877 | A/G | 0.318 | 0.300 | 0.305 | 0.297 | 0.287 | 0.287 | 0.289 | 0.288 |  | 224/983/1041 | 0.44 | 0.43 | 0.73 |
| **Note:** The table shows SNPs with P<0.05 in the replication phase. SNP, Single Nucleotide Polymorphisms; A1/A2, Minor/ Major allele; MAF, minor allele frequency; NSCL/P, non-syndromic cleft lip with or without palate; NSCLP, non-syndromic cleft lip with cleft palate; NSCLO, non-syndromic cleft lip only; BCLO, bilateral cleft lip only; UCLO, unilateral cleft lip only; RCLO, right cleft lip only; LCLO, left cleft lip only; HWE, Hardy-Weinberg equilibrium; GENO, genotype；O(HET)，Observed heterozygote frequency；E(HET)，Expected heterozygote frequency；P_HWE_, P-value for Hardy-Weinberg equilibrium test . | | | | | | | | | | | | | | |

| **Supplementary Table 6** Annotation of SNPs by HaploReg | | | | | | | |
| --- | --- | --- | --- | --- | --- | --- | --- |
| SNP | Chr | Position (Hg19) | Alt | Motifs changed | eQTL results | GENCODE genes | dbSNP function annotation |
| rs77179923 | 1 | 94097214 | T | 4 altered motifs |  | ABCA4 | intronic |
| rs12071152 | 1 | 94127843 | A | 7 altered motifs | 4 eQTL results | 6.7kb 5' of ABCA4 |  |
| Note: SNP, single nucleotide polymorphism; Chr:chromosome; Alt:alternate allele; eQTL:expression Quantitative Trait Loci. | | | | | | | |

| **Supplementary Table 7** Regulatory motifs altered by rs12071152 | | | | | |
| --- | --- | --- | --- | --- | --- |
| Position Weight Matrix ID (Library from Kheradpour and Kellis, 2013) | Strand | Ref | Alt | Match on: |  |
|  |  |  |  | Ref: TTTTGGACCTCAGGTTGTTGTTACTATTG | GATTGACATGTAGATATTGAAGTTTAGCTT |
|  |  |  |  | Alt: TTTTGGACCTCAGGTTGTTGTTACTATTG | AATTGACATGTAGATATTGAAGTTTAGCTT |
| DMRT5 | + | 11.8 | 14 | DWTTGWWACWKTKK | M |
| HNF1_6 | - | 11.1 | 12.9 | WDGTTAMTDWTY | WDYNNH |
| Irx | + | 12.3 | 13.5 |  | WDDNWACATGTWNDWNW |
| Mrg_2 | - | 12.7 | 12.4 | N | BWWTGACAGSTVNHW |
| Pbx-1_2 | - | 12 | 0.1 | WDWTT | GATTGATKDK |
| Pou5f1_disc1 | - | 13 | 14.2 |  | YWTTSWYATGCARAW |
| Pou5f1_known1 | + | 13.3 | 13.2 |  | YWTTSWYATGCWRAT |
| Sox_3 | - | 8.8 | 14.1 | MHNWAWTS | AATWRNHD |
| Note:Ref:reference allele; Alt:alternate allele. | | | | | |


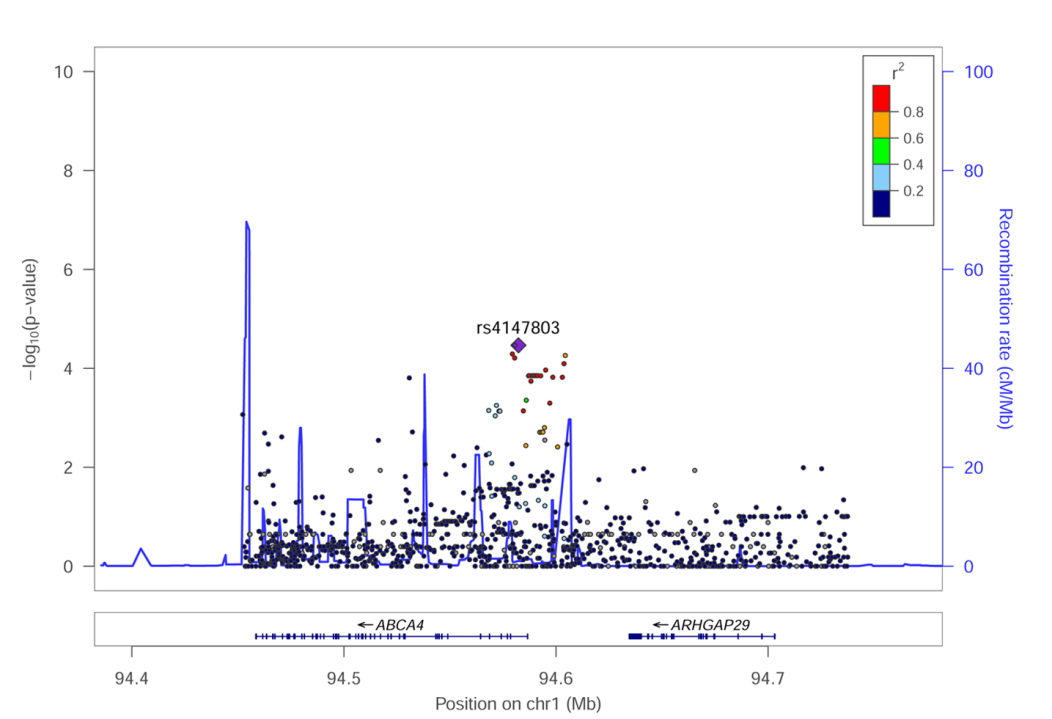


**Supplementary Figure 1**. LD Plots of SNP from association analysis of NSCL/P.


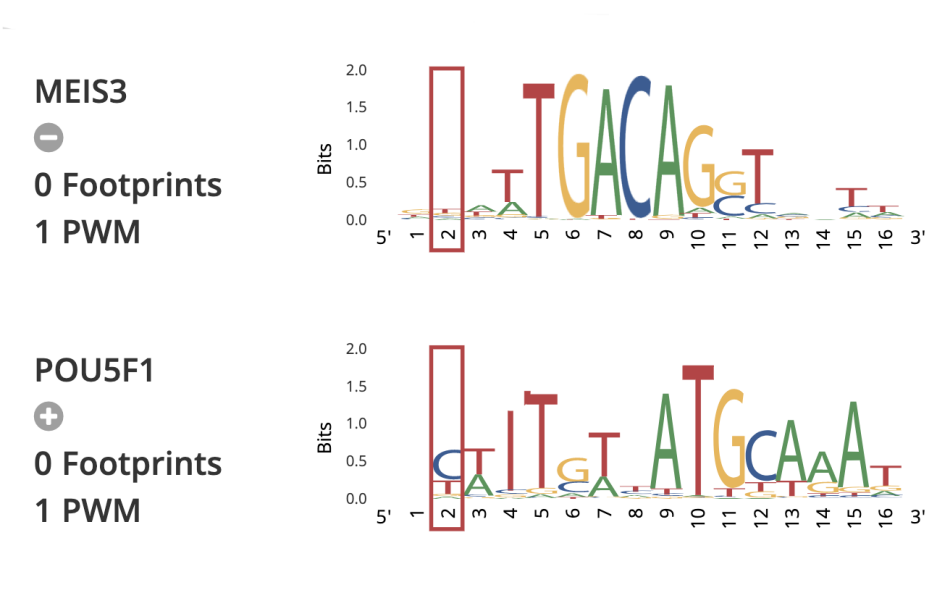


**Supplementary Figure 2**. Altered Motif of rs12071152 from RegulomeDB 2.0.3
